# An anti-CD103 antibody-drug conjugate prolongs the survival of pancreatic islet allografts in mice

**DOI:** 10.1038/s41419-019-1980-8

**Published:** 2019-09-30

**Authors:** Da Xue, Pili Liu, Wangming Chen, Chi Zhang, Lei Zhang

**Affiliations:** 1 Department of General Surgery, the Second Affiliated Hospital of Harbin Medical University, 246 Xuefu Road, 150086 Harbin, Heilongjiang Province China; 2 Department of Biology, Brandeis University, 415 South Street MB 1745, Waltham, MA USA 02453

**Keywords:** Allotransplantation, Type 1 diabetes, Experimental models of disease

## Abstract

CD103 mediates T-cell infiltration and organ allograft rejection, and depletion of CD103-expressing cells is a promising therapeutic strategy for allograft intolerance. Recently, we verified that M290-MC-MMAF, an anti-CD103 antibody-drug conjugate, potently eliminates CD103-positive cells in vivo, with high specificity and minimal toxicity. However, the contribution of M290-MC-MMAF to blocking the CD103/E-cadherin pathway involved in transplant rejection remains unclear. Herein, we examined the impact of systemic administration of M290-MC-MMAF on allografts in an islet transplantation model. M290-MC-MMAF treatment maintained the long-term survival of islet allografts (>60 days) compared to mock injection or unconjugated M290 antibody treatment (<18 days). The change was associated with a decrease in CD103^+^CD8^+^ effector T cells and an increase in CD4^+^CD25^+^ regulatory T cells. CD103^+^CD8^+^ effector T-cell transfer or CD4^+^CD25^+^ regulatory T-cell depletion resulted in a rapid loss of allografts in long-surviving islet hosts. Moreover, M290-MC-MMAF treatment reduced IL-4, IL-6, and TNF-α expression levels and increased IL-10 expression in the grafts, which presented an immunosuppressive cytokine profile. In conclusion, targeting CD103 with M290-MC-MMAF induced immunosuppression and prolonged the survival of pancreatic islet allografts in mice, indicating the potential clinical value of M290-MC-MMAF in therapeutic interventions for allograft rejection.

## Introduction

Type 1 diabetes is caused by the destruction of insulin-producing β cells by the autoimmune system. Endocrine replacement therapy via islet transplantation represents a viable and attractive therapeutic approach for treating type 1 diabetes^1^. Despite the improvement of allogeneic islet engraftment using systemic immunosuppression, this approach renders the recipients vulnerable to opportunistic infection and tumors. Thus, identifying more efficient and less harmful strategies to protect islet grafts is critical for improving islet transplantation outcome.

Alloreactive CD8^+^ T effector cells play key roles in allograft rejection by recognizing MHC-I/peptide complexes^2^. A subset of CD8 effector cells express α_E_(CD103)β_7_ (hereinafter referred to as CD103)^3,4^, an integrin that directs cells to the epithelial cell-specific ligand E-cadherin^5^. Through the interaction of CD103 and E-cadherin, CD103-expressing CD8 effector cells infiltrate and accumulate in the epithelial compartment of rejected renal allografts in mice^3^. This subset of CD8 effector cells has also been detected at the site of clinical renal allograft rejection^4^. Furthermore, CD103-deficient mice are phenotypically normal^6^, and the majority of pancreatic islet allografts survive indefinitely in these mice^7^. Moreover, CD103-deficient CD8^+^ cells are not retained and do not degrade the host intestinal epithelium in a mouse model of graft-versus-host disease^8^. These findings demonstrate a causal role of CD103^+^CD8^+^ effector cells in the destruction of grafted epithelial tissues and suggest an approach to induce allograft acceptance through the inactivation or disruption of CD103^+^CD8^+^ effector cells.

The therapeutic potential of CD103 blockade in organ transplantation has been assessed in several transplant models. In a rat model of allogeneic renal transplantation, treatment with an anti-CD103 monoclonal antibody (mAb) impeded the infiltration of CD8 effector cells into a renal epithelium graft and attenuated tubular injury without depleting CD103^+^CD8^+^ effector cells^9^. However, such nondepleting anti-CD103 mAbs fail to prolong the survival of tissue allografts. Hence, an anti-CD103 mAb (M290) was conjugated to various cytotoxins to achieve efficient depletion of CD103-expressing cells in vivo^10,11^. For example, M290-saporin (M290-SAP) has been demonstrated to deplete CD103-expressing leukocytes and promote indefinite survival of pancreatic islet allografts in MHC-mismatched mice^10,12^. Unfortunately, studies have shown that M290-SAP also depletes non-CD103-expressing T cells and causes significant weight loss^11^. As such, systemic toxicity restricts its clinical application. Our group previously synthesized three M290 antibody-drug conjugates (M290-ADCs) that efficiently and specifically depleted CD103-expressing cells in vivo without systemic toxicity^11^. In the present study, to evaluate the potential of the M290-ADCs in the therapeutic intervention of organ allograft rejection, one of the previously reported M290-ADCs (M290-MC-MMAF) was administered to streptozotocin (STZ)-induced diabetic mice with islet allograft transplantation. Our data support the hypothesis that the depletion of CD103-expressing cells is a promising therapeutic strategy for the management of islet allograft rejection.

## Materials and methods

### Animals

Male 6- to 8-week-old C57BL/6 (H-2^b^) recipients and BALB/c (H-2^d^) donor mice were purchased from Beijing HFK Bioscience Co., Ltd. (Beijing, China) and maintained under standard pathogen-free housing conditions with free access to food and water at the Harbin Medical University, Harbin, Heilongjiang. All mouse studies were performed in compliance with the Institutional Animal Care and Use Committee (IACUC) policies and guidelines at Harbin Medical University.

### Antibodies and ADCs

The M290 (rIgG2a) antibody against mouse CD103 was purchased from BioXCell (West Lebanon, NH, USA). Purified antibodies were sterile-filtered and stored at 4 °C in PBS. Maleimidocaproyl-monomethyl auristatin F (MC-MMAF) was synthesized by Concortis Biosystems Co., Ltd. (San Diego, CA, USA) as previously described^13^. The conjugate was prepared according to a previously published method^11,14^. The purity of M290-MC-MMAF was determined by size exclusion chromatography-high-performance liquid chromatography (SEC-HPLC)^11^.

### Safety evaluation of M290-MC-MMAF in mice

Wild-type (WT) C57BL/6 mice (6–8-week-old males) were intraperitoneally (i.p.) injected with M290-MC-MMAF (3 mg/kg) or the same dose of unconjugated M290 and/or PBS on days 1, 3, and 5. The clinical symptoms were observed. On day 10, the thymuses, livers, kidneys, and intestines of the treated mice were collected and fixed in 4% paraformaldehyde. Tissue specimens were then embedded in paraffin, sectioned, and stained with hematoxylin and eosin (H&E).

### Islet isolation and transplantation

Recipient mice were i.p. injected with 200 mg/kg streptozotocin (STZ) (Sigma-Aldrich, St. Louis, MO, USA) to induce diabetes. After 3 days, mice with a blood glucose level of >350 mg/dl for two consecutive days were used for transplantation.

Donor islets were isolated by a collagenase digestion method as described previously^15^. The pancreas was perfused with 2 mg/ml collagenase V (Sigma-Aldrich, St. Louis, MO, USA) and digested at 37 °C for 14 min. After digestion, the mixed pancreatic tissue was filtered with a 100-μm cell strainer (BD, USA), and purified islets were obtained by hand-picking under a stereoscope (SZM0745-B1; Aomei Co. Ltd., Nan Chang, China). Approximately 500 islets were transplanted under the left kidney capsule of the diabetic C57BL/6 recipient. After recovery from anesthesia, blood glucose levels in tail vein were monitored 2–3 times per week. Blood glucose levels of less than 200 mg/dl by day 3 after transplantation defined primary graft function, whereas blood glucose levels greater than 200 mg/dl for 2 consecutive days defined allograft dysfunction and loss^10^. Recipient C57BL/6 mice received three i.p. injections of 3 mg/kg M290-MC-MMAF or the same dose of unconjugated M290 and/or PBS on days 1, 3, and 5 post transplantation.

### Histopathology

On day 15 post transplantation, the islet graft and homolateral kidney were fixed and embedded with paraffin (*n* = 6 per group). The sections (5 μm thick) were routinely stained with H&E. To visualize the islets beneath the kidney capsule, a monoclonal anti-insulin antibody (clone K36aC10, Abcam, Cambridge, UK) was used for immunohistochemical staining in selected recipients. All captured images were assessed in a double-blind manner.

### Lymphocyte isolation

Cells were isolated from allografts, spleens, and mesenteric lymph nodes (MLN) by mincing the tissue with forceps and passing the resulting homogenate through a 40-μm BD cell strainer. Lymphocytes were further purified by centrifugation on a Lympholyte-M (CEDARLANE Laboratories Ltd., Burlington, NC, USA) to remove red blood cells. A TruCOUNT tube (BD Bioscience, San Jose, CA, USA) was used to calculate the absolute number of cells in a specific subpopulation.

### Flow cytometry

Flow cytometry was performed using a FACSCalibur (BD bioscience), and data were analyzed using FlowJo (TreeStar, Inc., Ashland, OR, USA) software. The percentage of positive cells for a given marker was based on cutoff points chosen to exclude >99% of the negative control population. For in vitro use, fluorochrome-conjugated mAbs specific for mouse CD103 (M290), CD4 (GK1.5), CD8a (53–6.7), CD3e (145–2C11), CD45 (30-F11), CD25 (3C7), and CD11c (HL3) and the respective species- and isotype-matched negative control mAbs were purchased from BD PharMingen (San Diego, CA, USA). A fluorochrome-conjugated mAb specific for mouse FoxP3 (FJK-16s) and isotype-matched negative control mAb were purchased from eBiosicence (San Diego, CA, USA).

### Cytotoxicity and lymphocyte proliferation assay in vitro

Splenic lymphocytes from WT C57BL/6 mice were plated at a final concentration of 1 × 10^6^ cells/well in 24-well plates with RPMI-1640 medium containing 10% FBS The lymphocytes were cultured with different concentrations of M290-MC-MMAF or without any treatment for 48 h at 37 °C in 95% O_2_ and 5% CO_2_. The collected cells were stained, and cell viability was analyzed based on 7AAD exclusion. The percentages of CD4^+^ and CD8^+^ T cells among gated CD3^+^ T cells were calculated. Apoptosis and necrosis were also evaluated with a Dead Cell Apoptosis Kit with Annexin V FITC and propidium iodide (PI) (Thermo Fisher Scientific, Eugene, OR, USA).

For the lymphocyte proliferation assay, 20 million carboxyfluorescein succinimidyl ester (CFSE) (Thermo Fisher Scientific, Carlsbad, CA, USA) labeled lymphocytes were stimulated with concanavalin A (ConA) (4 µg/ml) (Sigma-Aldrich, St. Louis, MO, USA) for 48 h, and then the cells were treated without or with M290-MC-MMAF (1 μg/ml or 10 μg/ml). After the cells were collected, the CFSE dilution was assessed in CD4 and CD8 subpopulations as a measure of proliferation.

### Quantitative real-time PCR (qRT-PCR)

RNA was extracted from the graft site using an RNApure™ Total RNA Extraction Kit (BioTeke, Beijing, China) and transcribed to cDNA using Super M-MLV reverse transcriptase (BioTeke). The levels of interleukin (IL)-4, IL-6, IL-10, and tumor necrosis factor-α (TNF-α) mRNAs were measured by quantitative real-time PCR on an Exicycler™ 96 Thermal Block (BIONEER, Daejeon, Korea) using SYBR Green Master Mix (Solarbio, Beijing, China) and the primers listed in Table 1. The relative expression levels were calculated by the 2^−ΔΔCt^ method, whereby β-actin served as the internal reference.

**Table 1:** RT-PCR primer sequences

Cytokine	Forward primer (5′ → 3′)	Reverse primer (5′ → 3′)
IL-4	TTGTCATCCTGCTCTTCTTTCT	ATGGCGTCCCTTCTCCTGT
IL-6	TGTATGAACAACGATGATGCAC	CTGGCTTTGTCTTTCTTGTT
IL-10	GAAGACAATAACTGCACCCACT	ACCCAAGTAACCCTTAAAGTCC
TNF-α	AGAAAGCATGATCCGCGAC	TTGTGAGTGTGAGGGTCTGG
β-actin	CTGTGCCCATCTACGAGGGCTAT	TTTGATGTCACGCACGATTTCC

### CD8^+^ T-cell adoptive transfer assay

For the adoptive transfer of CD8 cells into C57BL/6 hosts with long-surviving BALB/c allografts, WT or M290-MC-MMAF-treated mice (6-week-old males) that were to be used as lymphocyte donors were i.p. injected with 10 × 10^6^ BALB/c splenocytes. On day 6, the spleens and abdominal lymph nodes were harvested and centrifuged on Lympholyte-M to remove RBCs. The resulting cell suspension was enriched for CD8^+^ T cells by immunomagnetic bead sorting (MagniSort™ Mouse CD8 T cell Enrichment Kit, Thermo Fisher Scientific, Carlsbad, CA, USA). FACS analyses using anti-CD8, anti-CD3, and anti-CD103 antibodies were performed on each preparation to assess the degree of enrichment for CD8^+^ T cells and the expression of CD103. A total of 50 × 10^6^ CD8^+^ T lymphocytes were injected into C57BL/6 mice bearing long-surviving BALB/c islets via the tail vein, and blood glucose was monitored daily to assess graft survival.

### CD25^+^ T-cell depletion in vivo

Selected mice with long-surviving allografts (*n* = 6) were i.p. injected with 500 μg of purified CD25 mAb (PC61.5.3, Bio X Cell, West Lebanon, NH, USA) to deplete CD25^+^ T cells on day 60 post transplantation, as previously described^16^. The depletion was confirmed using the 7D4 antibody (BD PharMingen, San Diego, CA, USA), which recognizes a different epitope of CD25 than PC61.5.3 Ab, to stain splenic lymphocytes on day 7. Groups of mice were also i.p. injected with 500 μg of an isotype IgG (*n* = 5) or PBS (*n* = 6) on day 60 post transplantation as a control. The blood glucose levels of the recipients were monitored to evaluate the survival of the islet allografts.

### Statistical analysis

All data were analyzed using GraphPad Prism 8 software (GraphPad Software Inc., La Jolla, CA, USA) and presented as the mean ± SD. Statistical analyses were performed using the log-rank (Mantel−Cox) test to analyze allograft survival. ANOVA with Tukey’s multiple comparisons test was used to compare the differences among multiple groups, and unpaired Student’s *t* test was used for comparisons between two groups. *P* *<* 0.05 was considered indicative of statistical significance.

## Results

### M290-MC-MMAF treatment decreased CD8^+^ T-cell numbers and inhibited CD8^+^ T-cell proliferation in vitro

**Fig. 1:**
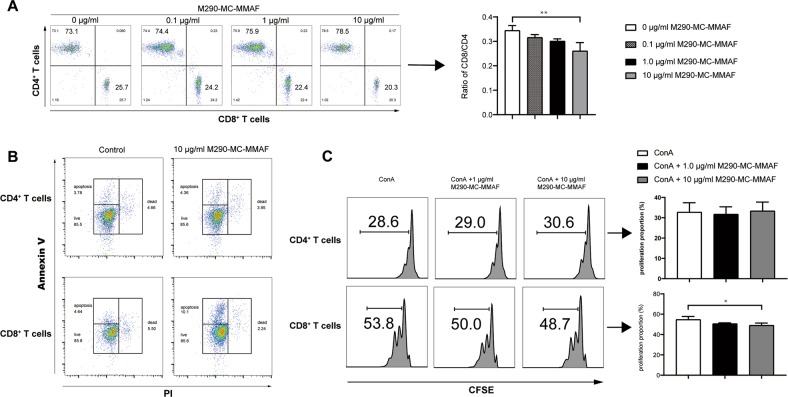
M290-MC-MMAF reduced and inhibited the proliferation of CD8^+^ T cells in vitro. **a** Splenic lymphocytes from C57BL/6 mice were treated with M290-MC-MMAF at the indicated concentrations for 48 h. The cells were subjected to multicolor FACS analyses using mAbs specific for CD3, CD4, and CD8. Data shown are the mean ± SD, *n* = 4 independent experiments. **b** Splenic lymphocytes from C57BL/6 mice were cultured with M290-MC-MMAF at the indicated concentrations for 32 h. FACS analyses were performed to determine the apoptosis/death of the CD4^+^ and CD8^+^ compartments using a Dead Cell Apoptosis Kit. Live cells, apoptotic cells, and dead cells are indicated. The data shown are representative of at least three independent experiments. **c** After stimulation with ConA (4 μg/ml) and treatment with M290-MC-MMAF at the indicated concentrations for 48 h, the proliferation of viable CD4^+^ or CD8^+^ compartments was determined. The CFSE^low^ population indicates proliferating cells. Data are the mean ± SD, *n* = 5 independent experiments. *P *<* 0.05, **P *<* 0.01, one-way ANOVA with Tukey’s multiple comparisons test

We performed in vitro experiments to evaluate the killing specificity of M290-MC-MMAF for CD103-expressing cells. As shown in Fig. 1a, the proportion of CD4 cells increased while the proportion of CD8 cells decreased; subsequently, the ratio of CD8^+^/CD4^+^ cells decreased significantly, demonstrating the high specificity of M290-MC-MMAF. Additionally, increased apoptosis in the CD8 compartment but not the CD4 compartment indicated the possible killing of CD103-expressing cells by M290-MC-MMAF (Fig. 1b). As shown in Fig. 1c, ConA-stimulated CD8^+^ T cells treated with M290-MC-MMAF demonstrated a reduced proliferative capacity. Moreover, the inhibition of CD8^+^ T-cell proliferation was positively correlated with the titrated concentration of ADC. In contrast, the response of CD4^+^ T cells was unimpaired under the same conditions. Taken together, these data are consistent with those of our previous study showing that M290-MC-MMAF specifically targets CD103, which is mainly expressed in the CD8 compartment.

### M290-MC-MMAF promoted pancreatic islet allograft survival in diabetic recipient mice

**Fig. 2:**
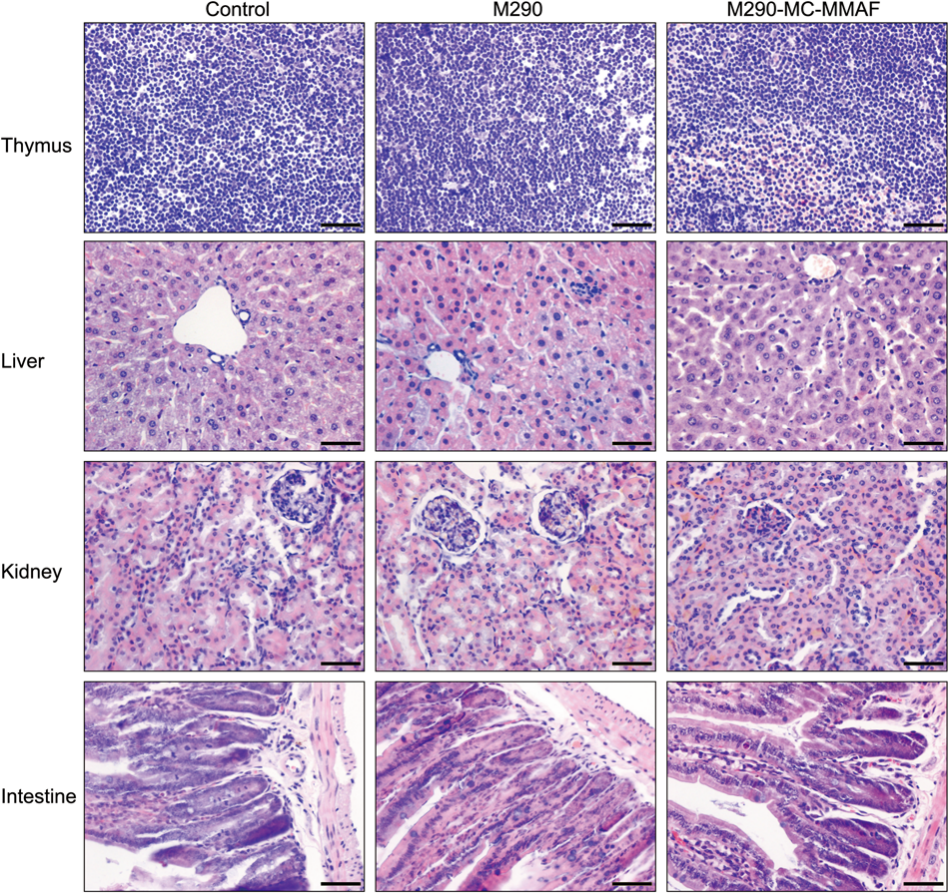
Safety of M290-MC-MMAF in vivo. Histopathological examination of murine tissues treated with M290-MC-MMAF (3 mg/kg) 10 days after the mice received three i.p. injections on days 1, 3, and 5. The photomicrographs do not reveal any evidence of toxicity from the M290-MC-MMAF and show a normal architecture similar to that of the PBS group and M290 group (*n* = 6 per group, Scale bars = 50 μm)

The potential toxicity of M290-MC-MMAF was evaluated 10 days after three i.p. injections of M290-MC-MMAF were administered to healthy C57BL/6 mice. The mice were normal and active after M290 or M290-MC-MMAF treatment. Histological micrographs of the thymus, liver, kidney, and intestine specimens collected from animals exposed to PBS, M290 or M290-MC-MMAF are presented in Fig. 2. There was no evidence of toxicity found in the specimens exposed to M290-MC-MMAF, and the results were found to be similar to those of the negative control group and M290 group. Our previous work demonstrated that M290-MC-MMAF did not cause weight loss in mice^11^. Here, we further show that M290-MC-MMAF is nontoxic in mice.

**Fig. 3:**
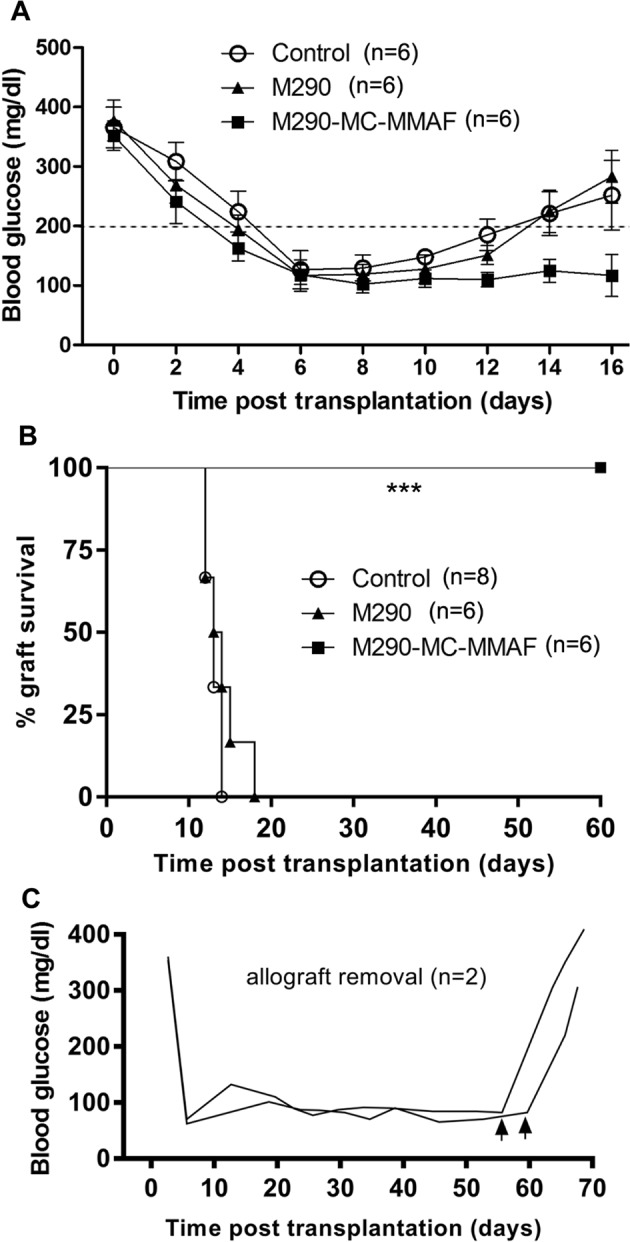
M290-MC-MMAF prolonged islet allograft survival in STZ-induced diabetic mice. Diabetic C57BL/6 recipients were transplanted with BALB/c islets and treated with three doses of M290-MC-MMAF (3 mg/kg i.p. injected) or the indicated controls on days 1, 3, and 5 post transplantation. **a** Blood glucose was monitored every other day post transplantation. The dashed line at 200 mg/dl is considered the threshold for allograft rejection. **b** Kaplan−Meier plots for graft survival. Graft survival was compared using the log-rank test (***P = 0.0001 vs. untreated). **c** Two M290-MC-MMAF-treated recipients developed hyperglycemia after removal of the graft on days 55 and 60 post transplantation

**Fig. 4:**
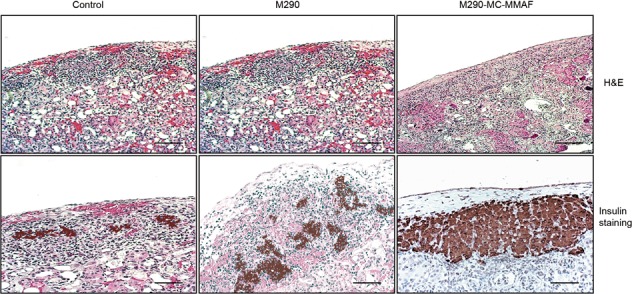
M290-MC-MMAF inhibited alloimmune inflammation and protected the function of insulin secretion. **a** The infiltration of leukocytes in islet grafts. Representative images of H&E staining of immune cells in paraffin-embedded allografts at 15 days post transplantation in untreated mice (control) and at 60 days post transplantation in long-surviving recipient mice. **b** Representative images of residual islet β cells in the renal capsular with immunohistochemical staining of insulin at 15 days post transplantation in untreated mice (control) and at 60 days post transplantation in long-surviving recipient mice (*n* = 6 per group, Scale bars = 100 μm)

BALB/c islet allografts were transplanted under the left renal capsule of STZ-induced diabetic C57BL/6 recipients. Islet allograft survival in the recipient mice was assessed. As shown in Fig. 3a, b, islet allograft rejection occurred within 18 days in the control and M290 groups. In contrast, systemic delivery of M290-MC-MMAF by three i.p. injections of 3 mg/kg markedly prolonged islet allograft survival (>60 days). Two of the mice developed hyperglycemia immediately after graft removal (Fig. 3c), indicating that it was the functional islet allograft that was responsible for blood glucose control in the recipients. H&E staining revealed that fewer immune cells infiltrated the islet allografts in M290-MC-MMAF recipients than the other two groups at 15 days post transplantation (Fig. 4a). Immunohistochemical staining with an anti-insulin antibody in formalin-fixed paraffin-embedded tissue graft sections showed more residual islets and an insulin-positive mass (Fig. 4b) in the grafts in M290-MC-MMAF-treated mice compared with the control recipients. Thus, M290-MC-MMAF prevented leukocyte infiltration and increased insulin secretion by islet allografts. These results indicate that M290-MC-MMAF-treated recipients have prolonged allograft survival compared with control-treated recipients.

### M290-MC-MMAF treatment altered T-cell subpopulations in allograft recipients

**Fig. 5:**
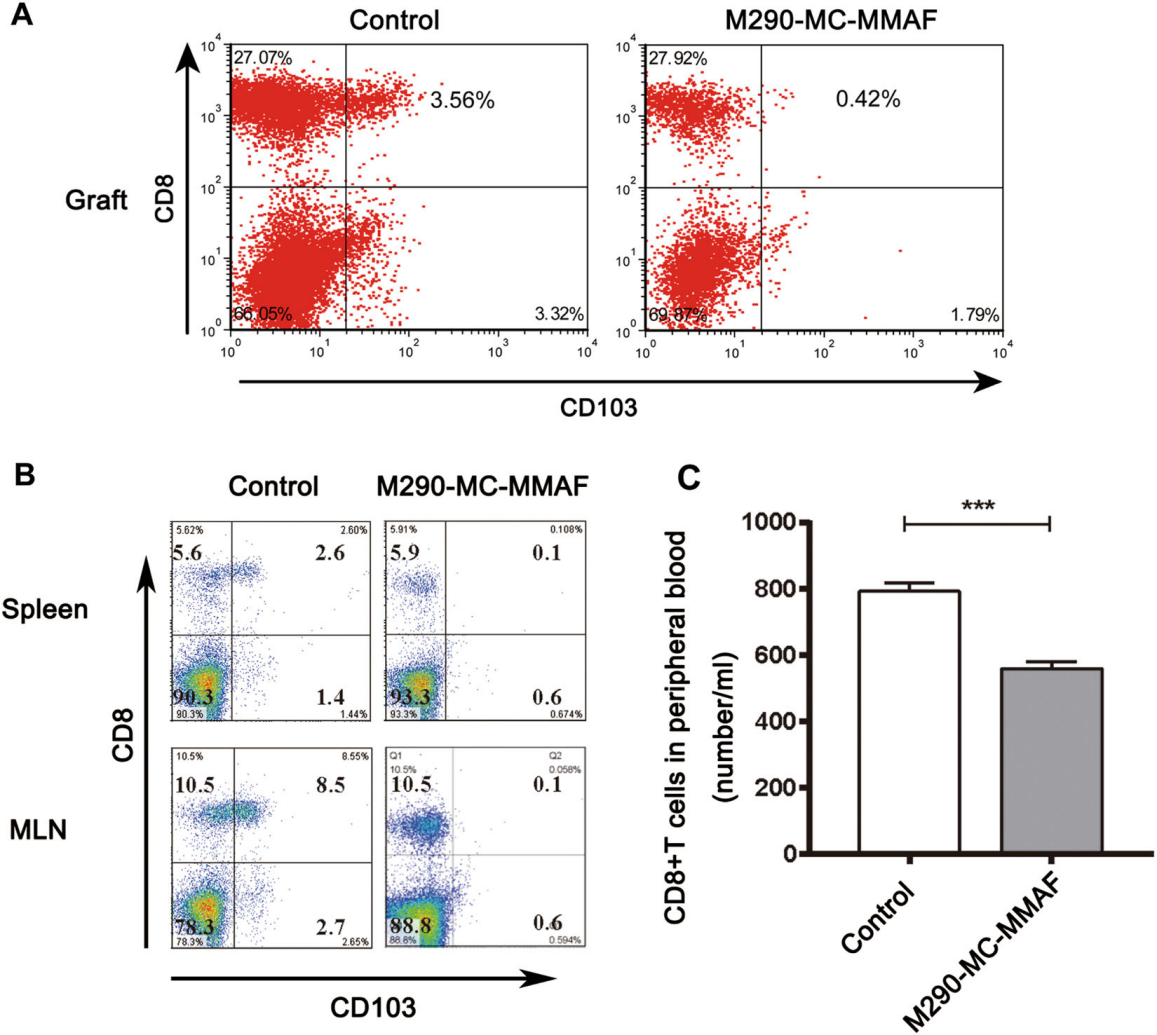
M290-MC-MMAF depleted CD103^+^CD8^+^ cells and led to lymphopenia in the CD8 compartment in the recipient mice. **a** CD103 expression in graft-infiltrating lymphocytes and **b** splenic and MLN CD8+ T cells were determined by FACS analyses at day 15 post transplantation. **c** The absolute number of CD8^+^ T cells in peripheral blood was calculated using TruCount beads. Data are presented as the mean ± SD (*n* = 7 per group). The shown percentages are representative data. MLN mesenteric lymph nodes. ***P *<* 0.001, unpaired *t* test

The frequencies of CD8^+^CD103^+^ cells in the grafts, spleen, and MLN of recipient mice with and without M290-MC-MMAF treatment were examined by flow cytometry 15 days after islet transplantation. As shown in Fig. 5a, b, M290-MC-MMAF treatment eliminated the CD8^+^CD103^+^ T-cell subpopulation in the graft, spleen, and MLN at the time when allograft rejection occurred in the other two recipient groups. Importantly, the absolute number of CD8^+^ T cells in the peripheral blood was also reduced by treatment with M290-MC-MMAF (Fig. 5c).

**Fig. 6:**
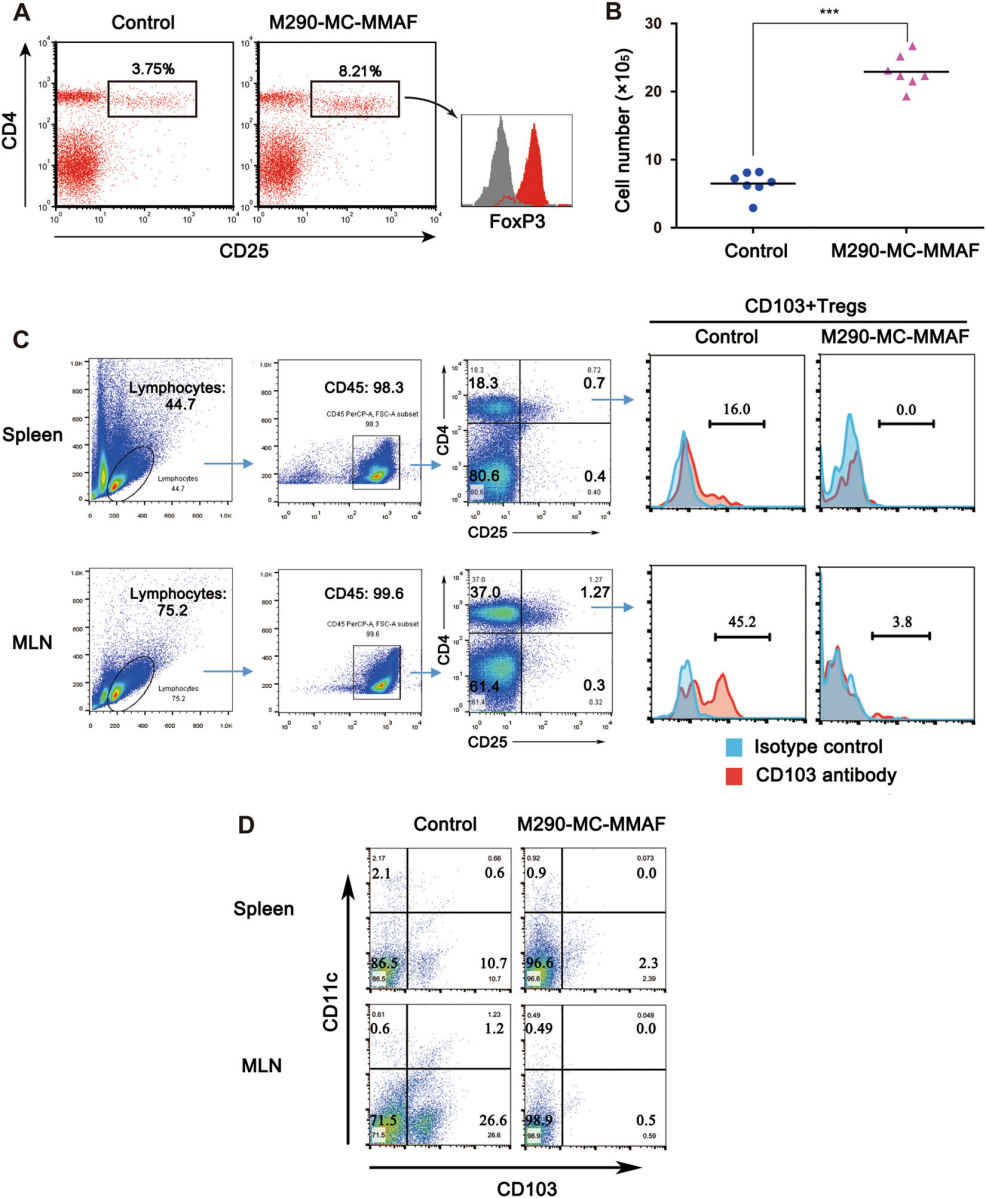
Foxp3^+^ Tregs and CD103^+^ DCs in recipient mice. **a** Proportions of splenic FoxP3^+^CD4^+^CD25^+^ Tregs were determined by FACS analyses at day 15 post transplantation in mock-treated control mice and at day 60 post-transplantation in long-surviving recipient mice. **b** Absolute number of CD4^+^CD25^+^ cells in (**a**). CD103 expression in the splenic and MLN CD4^+^CD25^+^ Tregs (**c**) and CD11c^+^ (**d**) cells were determined by FACS analyses at day 60 post transplantation in long-surviving recipient mice (*n* = 7 per group). The data shown are representative data. MLN mesenteric lymph nodes. ***P *<* 0.001, unpaired *t* test

For the Treg subpopulation, M290-MC-MMAF-treated recipients bearing long-surviving islet allografts exhibited significantly higher frequencies and absolute numbers of CD4^+^CD25^+^ cells in the spleen than the control recipients experiencing allograft rejection (Fig. 6a, b). Additionally, the CD4^+^CD25^+^ cells in the spleens of M290-MC-MMAF-treated recipients contained a significant number of FoxP3-expressing cells. However, the CD103^+^ Tregs and CD103^+^ dendritic cells (DCs) in the spleens and MLNs of recipients bearing long-surviving islet allografts were eliminated by M290-MC-MMAF treatment (Fig. 6c, d). These results imply a possible association of Treg expansion with prolonged allograft survival.

### M290-MC-MMAF-modulated local cytokine expression at the pancreatic islet allograft site

**Fig. 7:**
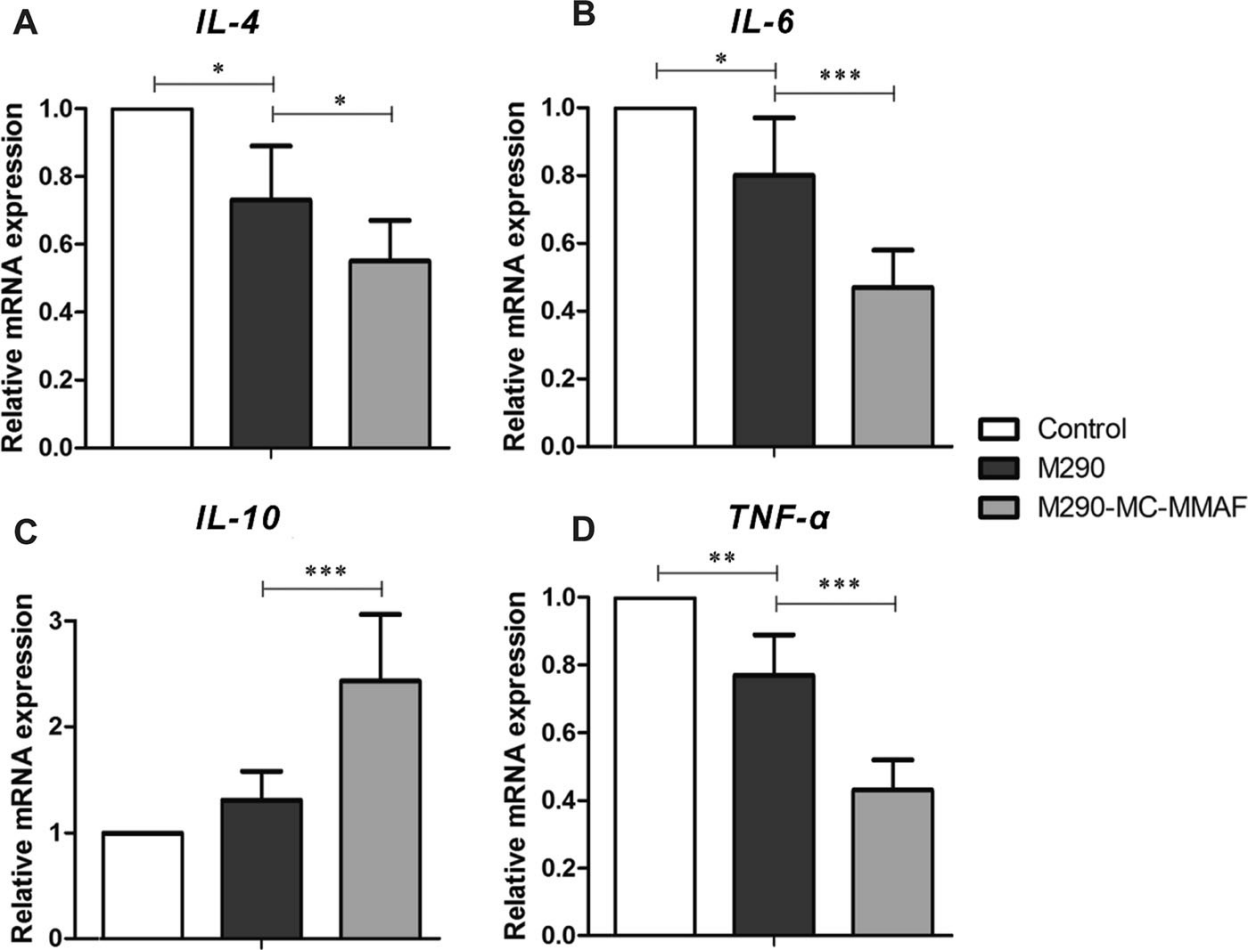
M290-MC-MMAF produced cytokine profiles favoring islet survival at the graft site. Relative levels of **a** *IL-4*, **b** *IL-6*, **c** *IL-10*, and **d** *TNF-α* mRNA in the grafts of three groups of recipients were quantified by qRT-PCR on day 15 post transplantation. Data are the mean ± SD (*n* = 6 per group); *P *<* 0.05, **P *<* 0.01, and ***P *<* 0.001, one-way ANOVA with Tukey’s multiple comparisons test

To evaluate the immunosuppressive effects of M290-MC-MMAF treatment in the pancreatic islet allografts, the expression of inflammatory cytokines at the graft site in recipient mice was determined 15 days after islet transplantation. As shown in Fig. 7, M290 treatment decreased the expression levels of *IL-4*, *IL-6*, and *TNF-α* in the grafts compared to the mock injection control treatment, while M290-MC-MMAF treatment further reduced the levels of *IL-4*, *IL-6*, and *TNF-α* and significantly increased the expression of the anti-inflammatory cytokine *IL-10* at the graft site. These data suggest that local M290-MC-MMAF-modulated cytokine expression may be involved in the suppression of allograft rejection in recipient mice.

### CD103^−^CD8^+^ T cells exhibited a defect in inducing islet allograft rejection

**Fig. 8:**
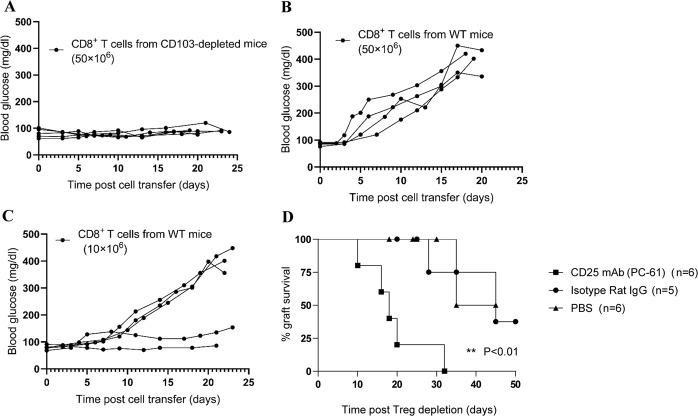
Adoptive CD103^+^CD8^+^ cell transfer or depletion of CD4^+^CD25^+^ Tregs resulted in loss of long-term surviving islet grafts in recipient mice. CD8^+^ T cells from CD103-depleted C57BL/6 mice (treated with M290-MC-MMAF) (**a**) or WT (**b**, **c**) were adoptively transferred into long-surviving recipients (>60 days). Donors were intraperitoneally immunized with 1× 10^7^ BALB/c splenocytes 4 days before cell isolation. Each line represents blood glucose levels from a single recipient mouse. CD8^+^ cells for these experiments were enriched by immunomagnetic beads. **d** An anti-CD25 mAb (PC-61; 500 μg) was i.p. injected into the long-surviving recipient mice (>60 days) to deplete CD25^+^CD4^+^ Tregs. Recipients were treated with rat IgG or PBS as a control. The Kaplan−Meier curve was plotted for graft survival. Graft survival was compared using the log-rank test (**P < 0.01 vs. PBS control)

Adoptive transfer experiments were used to determine whether the destruction of islet allografts by CD8^+^ T cells depends on CD103 expression. In these experiments, CD8^+^ T cells from WT or CD103-depleted mice (CD103-MC-MMAF treated) were enriched and transferred into C57BL/6 mice with long-surviving BALB/c islets. As shown in Fig. 8a, the transfer of highly enriched CD8^+^ T cells from CD103-depleted mice into hosts had no effect on islet allografts. In contrast, the transfer of the equivalent CD8^+^ T cells from WT mice elicited rejection (Fig. 8b). Additionally, this rejection seemed to be positively correlated with the number of transferred cells, since transferring a small number of CD8^+^ T cells led to only a partial loss of islet graft function in recipients (Fig. 8c). Taken together, these data suggest that CD103^−^CD8^+^ T cells lack the capacity to efficiently induce islet allograft rejection.

### Induction of islet allograft rejection after the deletion of CD25^+^CD4^+^ T cells

The frequencies and numbers of CD25^+^CD4^+^ cells in the spleen and islet transplant site in the recipients with long-surviving islet allografts were significantly higher than those in the control recipients undergoing allograft rejection. This result led us to hypothesize that CD25^+^CD4^+^ Tregs contribute to the long-term survival of islet allografts. To test this hypothesis, we i.p. injected 500 µg of anti-CD25 mAb (PC61.5) on day 60 post transplantation to remove CD25^+^CD4^+^ Tregs. As previously reported, PC61.5 eliminates the majority of CD25^+^CD4^+^ Tregs in the peripheral blood and spleen. All allografts were quickly rejected when the recipients were treated with anti-CD25 mAb (Fig. 8d). The survival of the allografts was significantly shorter in anti-CD25-treated mice than in IgG-treated mice and PBS-treated mice (*P* < 0.001). These results indicate that CD25^+^CD4^+^ Tregs are necessary to maintain the long-term functionality of islet allografts.

## Discussion

It is well documented that CD103 directs a subpopulation of CD8^+^ T cells to epithelium-specific E-cadherin^5^, resulting in the accumulation of this T-cell subpopulation in the epithelial compartment of rejected grafts^3,4^. Moreover, pancreatic islet allografts survive indefinitely in CD103-deficient mice^7^, and CD103-deficient CD8^+^ cells fail to accumulate and attack the host epithelium in a graft-versus-host disease model^8^. The crucial role played by CD103-expressing cells in allograft rejection has prompted attempts to target CD103 for the induction of graft tolerance^9,10,12^. Compared with the nondepleting CD103 Ab treatment, CD103-based immunotoxin (M290-SAP) treatment depletes CD103^+^CD8^+^ effector cells and prolongs the survival of pancreatic islet allografts in mice^10^. However, the systemic toxicity of M290-SAP (nonspecific T-cell depletion, severe thymus atrophy, ascites, and significant weight loss^10,11^) is likely attributable to the strong cytotoxicity of saporin^17^, compromising its clinical translational value. Thus, agents targeting CD103 with high specificity and low toxicity are required for the induction of immunosuppression after cell or solid organ transplantation. Previously, our group synthesized several M290-ADCs that are cytotoxic only after being internalized via CD103^11^. These M290-ADCs, including M290-MC-MMAF, show higher specificities than M290-SAP in targeting CD103-expressing cells in vivo^11^. In the present study, the potential toxicity of M290-MC-MMAF was further assessed, and no aberration was observed in the mouse thymus, liver, kidney, or intestine 10 days after M290-MC-MMAF administration. These data verify the safety of M290-MC-MMAF for in vivo applications.

The main purpose of the study was to evaluate the therapeutic potential of M290-MC-MMAF in islet transplantation. M290-MC-MMAF maintained the integrity and long-term functionality of the allogeneic pancreatic islets in diabetic mice. Two underlying mechanisms have been proposed to explain the observed islet graft survival associated with CD103-MC-MMAF treatment: (1) specific lymphopenia in the CD8^+^ T compartment and (2) a shift toward a Treg phenotype.

Our results demonstrate that CD103-MC-MMAF treatment depleted CD103^+^CD8^+^ cells in vitro (Fig. 1) and in vivo (Fig. 5). Both apoptosis and necrosis were involved in the decay of the CD8^+^ T cells (Fig. 1b). The capacity of CD8^+^ T cells to destroy islet allografts is highly dependent on CD103 expression and was established using adoptive transfer models. CD8 cells from WT mice induced the rejection of long-surviving allografts, whereas CD8^+^ cells from CD103-MC-MMAF-treated mice were completely ineffectual in this regard (Fig. 8a).

CD4^+^CD25^+^ Tregs play a key role in the maintenance of immunologic self-tolerance and the suppression of pathological immune responses through the contact-dependent suppression of T-cell proliferation and the control of inflammation via soluble cytokines^18–20^. CD103 is expressed in a subset of Tregs^21^, which raises the concern that CD103-based immunotoxins may decrease the Treg population and disrupt T-cell homeostasis, resulting in immunological chaos. In the present study, M290-MC-MMAF treatment resulted in a reduction in CD103^+^ Tregs in the early stage, but these changes did not affect the survival of the islet allograft, suggesting that CD103-expressing Tregs may not be overly important in the regulation of islet allograft rejection. Strikingly, M290-MC-MMAF-treated recipient mice with long-term surviving islet allografts had significantly higher percentages and absolute numbers of CD4^+^CD25^+^ cells in the spleen than untreated mice undergoing allograft rejection. Similar results were also observed in clinical data, which showed that increased frequencies of FoxP3^+^CD4^+^CD25^+^ Tregs 3 weeks after lung transplantation were associated with a reduced chance of chronic lung allograft dysfunction at 2 years^22^, suggesting that immediate expansion of FoxP3^+^CD4^+^CD25^+^ Tregs post transplantation may exert a protective effect on the long-term survival of the grafts. Removal of CD4^+^CD25^+^ Tregs from long-surviving allograft recipients treated with an anti-CD25 mAb led to accelerated rejection, supporting the assumption that the Tregs in the system and/or local graft environment protect islet allografts from the immune attack of cytotoxic CD8 cells. Parallel findings have shown that Tregs function in the control of immune responses after allo- and xenogeneic islet transplantation^23,24^ or solid organ transplantation^25,26^. However, it remains to be determined whether the elimination of CD103^+^CD4^+^CD25^+^ Tregs changes the homeostasis of total Tregs and leads to the expansion of total Tregs in M290-MC-MMAF-treated recipients, which is similar to the Treg proliferation caused by lymphocyte depletion using antibody therapy, such as the anti-CD3 antibody^27,28^ or Campath [anti-CD52]^16,29^.

CD103 is also expressed by antigen-presenting DCs^30,31^. Here, the number of CD103^+^ DCs in the spleen and MLN was substantially depleted by M290-MC-MMAF treatment, which raises the concern that, while unlikely, these CD103^+^ DCs are involved in prolonged graft survival. First, the proportion of CD103^+^ DCs in the spleen and MLN is very small (<1.5%). Second, the level of CD103 DCs of donor origin present in islet allografts is extremely low (even negligible). More importantly, eliminating the CD103-expressing DCs from donors by pretreatment with M290-SAP did not prolong allograft survival^10^. Considering that CD103^+^CD8^+^ effector T cells play causal roles in the destruction of grafts^7–9^, we believe that treatment with M290-MC-MMAF prolongs islet allograft survival mainly by depleting CD103-expressing CD8^+^ effector T cells. Overall, the present study has provided evidence that the dynamic equilibrium between CD8 effectors and Tregs is essential in preserving the long-term survival of islet grafts. Strengthening the attack on islet grafts or weakening the protection of islet grafts leads to the loss of graft function, which was verified in the cytotoxic CD8 T-cell adoptive transfer and CD4^+^CD25^+^ Treg depletion models (Fig. 8).

A variety of cytokines orchestrate the immunologic responses leading to graft tolerance or rejection. Posttransplant IL-4 treatment can convert liver allograft tolerance to rejection^32^. IL-6 and TNF-α synergistically impair Treg-mediated suppression of T effector alloimmunity and reduce the efficacy of therapies that promote allograft tolerance^33^. In contrast, IL-10, secreted by type I Tregs and macrophages, suppresses T-cell proliferation and induces long-term allograft survival^34,35^. In our study, M290-MC-MMAF treatment led to reduced expression of *IL-4*, *IL-6*, and *TNF-α* and dramatically increased the expression of *IL-10* in the grafts, presenting a tolerogenic phenotype. Notably, M290 treatment also reduced the expression of *IL-4*, *IL-6*, and *TNF-α* in the grafts, but to a lesser extent than M290-MC-MMAF treatment. This was probably because the temporary masking of CD103 by M290 delayed CD103^+^CD8^+^ T-cell infiltration into the graft and the consequent inflammatory reactions.

Collectively, the present study demonstrated that M290-MC-MMAF treatment prolongs the survival of pancreatic islet allografts in mice without causing any detectable toxicity. M290-MC-MMAF treatment not only decreased CD8^+^ T cells by depleting CD103^+^ cells but also increased the number of Tregs in the hosts and modulated cytokine production in the grafts. The present data suggest the potential value of M290-MC-MMAF treatment in the therapeutic intervention of allograft rejection, and further assessments, such as measurement of the optimal dose and administration time, are required for accelerated clinical conversion.
